# Genome wide analysis implicates upregulation of proteasome pathway in major depressive disorder

**DOI:** 10.1038/s41398-021-01529-x

**Published:** 2021-07-28

**Authors:** Shaked Belaish, Ifat Israel-Elgali, Guy Shapira, Israel Krieger, Aviv Segev, Uri Nitzan, Michael Majer, Yuval Bloch, Abraham Weizman, David Gurwitz, Noam Shomron, Libi Hertzberg

**Affiliations:** 1 Sackler Faculty of Medicine, Tel Aviv University, Tel Aviv, Israel; 2 Sagol School for Neuroscience, Tel Aviv University, Tel Aviv, Israel; 3 Edmond J. Safra Center for Bioinformatics, Tel Aviv University, Tel Aviv, Israel; 4 Shalvata Mental Health Center, Hod Hasharon, Israel; 5 Department of Physics of Complex Systems, Weizmann Institute of Science, Rehovot, Israel; 6 Geha Mental Health Center and Felsenstein Medical Research Cener, Petah Tikva, Israel

**Keywords:** Neuroscience, Depression

## Introduction

Major depressive disorder (MDD) is a complex and common psychiatric illness [1]. The World Health Organization predicts that by 2030, MDD will be the leading cause of disease burden worldwide (https://www.who.int/mental_health/management/depression/wfmh_paper_depression_wmhd_2012.pdf?Ua=1). Despite the numerous pharmacological agents available, about 30% of persons with MDD do not achieve a satisfying response from their medications [2]. Thus, the importance of improving therapy cannot be overestimated. However, to improve treatment options, a better understanding of the biology of MDD is needed. While the genetic contribution is estimated at around 45% [3], the biological basis of MDD is still poorly understood.

The most recently published MDD genome-wide association studies (GWAS) applied a meta-analysis to 135,458 cases and 344,901 controls and detected 44 MDD associated loci; each contributing only slightly to the risk to develop MDD [4]. Although the list of associated loci is given by GWAS, identifying the causal variants driving these associations is a complex task, for the following reasons: (i) Many loci span large numbers of genes due to the pattern of linkage disequilibrium [5]. In addition, a substantial portion of the loci resides inside promoters or enhancers that affect the expression of genes distant from the locus (similar to schizophrenia, for example [6]). Thus, it is difficult to pinpoint the causal variant inside a given associated locus and the gene associated with the causal variant. (ii) The biological interpretation of GWAS results remains challenging, particularly in the field of neuropsychiatric disorders, due to their polygenic nature and the small effect of single genetic factors [7,8,]. Several approaches have been developed to detect the potential causal genes (for example, the VEGAS tool [9], which translates GWAS results at the single nucleotide polymorphism (SNP)-level to the gene-level). However, focusing on a specific gene can be misleading, as it could represent a false-positive finding. Thus, a higher-level analysis, such as pathway enrichment analysis (PEA), is needed to improve the interpretation of results in relation to the biological pathways involved. (iii) Performing PEA based on MDD GWAS results alone is limited since the list of GWAS-based genes is not well-defined (as described in (i)) and might be too short to reach statistically meaningful PEA (as was demonstrated in [10]).

In our study, we integrated GWAS results with global gene expression data, to improve the interpretation of MDD GWAS results, in relation to the biological pathways involved. The integration of data from various sources may potentially decrease false-positive rates resulting from each data source separately, and increase the reliability of the results [11,12,]. We previously used this approach to decipher pathways involved in schizophrenia [10,13,]. In the first study [10], gene expression data were integrated with schizophrenia GWAS results to (i) identify a cluster of GWAS-based genes with highly correlated expression, and (ii) extend the cluster to include genes whose expression correlates highly with the cluster’s pattern. This enabled a statistically meaningful PEA of GWAS-based genes associated with schizophrenia, which could not be performed when analyzing GWAS-based genes alone. Similarly, we expected that the integration of GWAS with gene expression data would improve the interpretation of MDD GWAS results, in relation to the biological pathways involved.

We extracted 37 GWAS-based genes from the latest published GWAS of MDD [4]. We explored their expression patterns in post-mortem brain samples of 48 individuals with MDD (GSE53987) and identified a statistically significant pairwise correlation pattern. This suggests a possible biological basis for the observed correlations and involvement in common biological pathways. However, the PEA of the 37 GWAS-based genes did not yield reliable results. We then identified a cluster of seven highly correlated GWAS-based genes. We followed the rationale that genes participating in common biological pathways tend to have correlated expression [14], and expanded the cluster by 793 genes highly correlated with the cluster’s average profile. We were then able to apply statistically meaningful PEA and identified biological pathways that are known to be involved in brain-related processes and specifically in MDD. These results were replicated in an independent dataset. While gene-level differential expression analysis of the GWAS-based genes and the enriched pathways did not yield statistically significant results, pathway-based differential expression analysis did identify a differentially expressed pathway in MDD.

## Materials and methods

1. Data
  1. Gene expression data: Messenger RNA (mRNA) levels from post-mortem brain samples of individuals with MDD and healthy controls measured by microarrays were obtained from the Gene Expression Omnibus (GEO) repository [15]. We used three datasets, composed of six brain regions (484 samples overall), as listed in Table 1. Demographic and samples characteristics are presented in Table S1. The gene expression datasets used were pre-processed according to standard methods; for details see the Supplementary Methods section 2 and Fig. S1.

**Table 1:** Gene expression data description.

GEO accession	Brain region	# MDD samples	# Control samples	Gender distribution	Mean age	Platform
GSE53987 (Lanz et al., 2015)	Hippocampus (HPC)	16^a^	18	MDD—M = 9, F = 8 Control—M = 9, F = 9	MDD—45.17 Control—48.16	Affymetrix Human Genome U133 Plus 2.0
	Striatum (STR)	15^b^	18	MDD—M = 10, F = 6 Control—M = 10, F = 8	MDD—46.5 Control—48.44	
	Brodmann area 46 (BA46)	17	19	MDD—M = 9, F = 8 Control—M = 10, F = 9	MDD—45.17 Control—48.05	
GSE35978 (Chen et al., 2013)	Parietal cortex (PC)	14	51	MDD—M = 8, F = 6 Control—M = 35, F = 15	MDD—46.07 Control—45.5	Affymetrix Human Gene 1.0 ST Array
	Cerebellum (CRBLM)	13	50	MDD—M = 8, F = 5 Control—M = 31, F = 19	MDD—45.38 Control—45.8	
GSE92538 (Hagenauer et al., 2018)	The dorsolateral prefrontal cortex (DLPFC)	76	175	MDD—M = 60, F = 16 Control—M = 130, F = 45	MDD—48.6 Control—55.9	Affymetrix GeneChip Human Genome HG-U133 Plus 2 Array

^a^One sample was removed from the hippocampus (HPC)-MDD (GSE53987) as it did not pass the quality control (see Fig. S1A). Of 17 samples, we continued to investigate the remaining 16.

^b^One sample was removed from the striatum (STR)-MDD (GSE53987) as it did not pass the quality control (see Fig. S1B). Of 16 samples, we continued to investigate the remaining 15.
  2. GWAS data: Results were obtained from the latest MDD GWAS study (135,458 cases and 344,901 controls), in which the genes located 200 kb downstream and upstream to each of the 44 resulting associated loci were listed [4]. We created a list of 37 GWAS-based genes, which includes the closest gene (within 200 kb downstream and upstream) to the peak SNP of each of the 44 associated loci.
  3. Blood sample data: The research protocol was approved by the Ethics Committee of Shalvata Mental Health Center, and all the participants provided written informed consent. Nine patients with MDD and nine healthy controls were recruited from Shalvata Mental Health Center, Israel. All the patients had been diagnosed with MDD according to the Diagnostic and Statistical Manual of Mental Disorders (DSM)-IV or DSM-V. Current depressive symptoms were analyzed using the Hamilton Depression Rating Scale (HAM-D) [16] and the Quick Inventory of Depressive Symptomatology (QIDs) [17]. Peripheral blood samples were obtained at baseline. Demographic and samples characteristics of the participants are presented in Table S2.
2. Expression correlation analysis of GWAS-based gene lists:
  For a given gene list, expression pairwise Pearson correlation coefficients were calculated along with the MDD samples, for each region separately. For each correlation value, a *p*-value was calculated using the MATLAB function “corr“, which calculates the probability of obtaining such an absolute value of the correlation, or higher, in a random permutation of the order of the samples.
3. Calculation of a *p*-value of the observed correlation pattern:
  We calculated the pairwise gene expression correlation values of a randomly selected group of genes of the same number as the given GWAS-based gene list. The random group of genes was created using a uniform distribution on the whole gene expression dataset. We repeated this calculation 1000 times. To calculate the p-value of the observed correlation pattern, we counted the number of random groups in which the number of gene pairs with absolute correlation values higher than a certain threshold is equal to or higher than that of the given GWAS-based gene list. For a detailed description see Supplementary Methods section 3.
4. Extending the list of GWAS-based genes for PEA:
  Given pairwise Pearson correlation values of the expression patterns of a list of GWAS-based genes, we first identified a cluster of genes with highly correlated expression. Then we calculated the average expression profile of the cluster and extended the cluster that originated from the GWAS-based genes to 800 genes in total. We did this by adding the genes with the highest Pearson correlation to the cluster’s average expression profile, as calculated along with all the MDD samples of the relevant brain region. Extending the list is important for enabling robust and statistically meaningful PEA.
5. PEA using GeneAnalytics via the GeneCards Suite website:
  Given a group of genes, we applied the GeneAnalytics tool [18] to identify enrichment of biological pathways. GeneAnalytics defines superpathways as comprising one or more pathways from different data sources, based on the similarity of their compound genes. This entity was established to improve inferences and to reduce redundancy. Superpathway enrichment scores are based on log2-transformation of the binomial *p*-value, which is equivalent to a p-value corrected for multiple comparisons, with significance defined at <0.05. According to GeneAnalytics, scores are classified as: High: corrected *p*-value smaller or equal to 0.0001; Medium: corrected *p*-value higher than 0.0001 but lower or equal to 0.05; Low: corrected *p*-value higher than 0.05.
6. Estimation of the comparability of various datasets and brain regions, according to the differential expression:
  We used an accepted measure to assess the comparability of two datasets, as was presented in [19]. First, a two-sided *t*-statistic was calculated for each gene, comparing its expression levels between individuals with MDD and controls, for each brain region separately. Then, to assess the comparability of two given brain regions (from the same or different datasets), Pearson correlation between the *t*-statistic values along all the genes expressed in both regions was calculated. A *p*-value for obtaining such a correlation value (or higher, in terms of absolute value) was also calculated.
7. Pathway-based differential expression analysis using the STRING database:
  Network creation: A network is defined by a given group of genes (nodes) and the co-expression relations of the genes (edges). We used the STRING database, version 10.5 [20] for the co-expression relations data. Accordingly, a score between 0 and 1 “indicates the estimated likelihood that a given interaction is biologically meaningful, specific and reproducible” [20]. Edges with a score above 0.1 were included.
  Network view of differential expression: Given a gene expression dataset, the following calculation was applied for each gene in the network:
  - The mean expression and standard deviation values, Mc and Sc, are calculated using the control samples only.
  - The mean expression, Mp, is calculated using the MDD samples.
  - Mp-Mc is calculated as the difference in the mean expression between the two groups of samples.
  - The deviation from the control group is calculated, by (Mp − Mc)/Sc. Then the network is displayed as an undirected graph such that the node’s colors correspond to the deviation described above, (Mp − Mc)/Sc. The edges represent co-expression relations. Only genes that have co-expression relations with other genes in the network are displayed.
8. RNA-sequencing of peripheral blood mononuclear cells:

Peripheral blood mononuclear cells (PBMC) were extracted from the samples collected at Shalvata Mental Health Center (9 MDD and 9 controls). RNA was then extracted and sequenced. For a detailed description, see the Supplementary Methods section 10.

## Results

### MDD GWAS-based gene expression exhibits a high pairwise correlation

A list of 37 GWAS-based genes was created, based on the latest MDD GWAS [4].

**Fig. 1:**
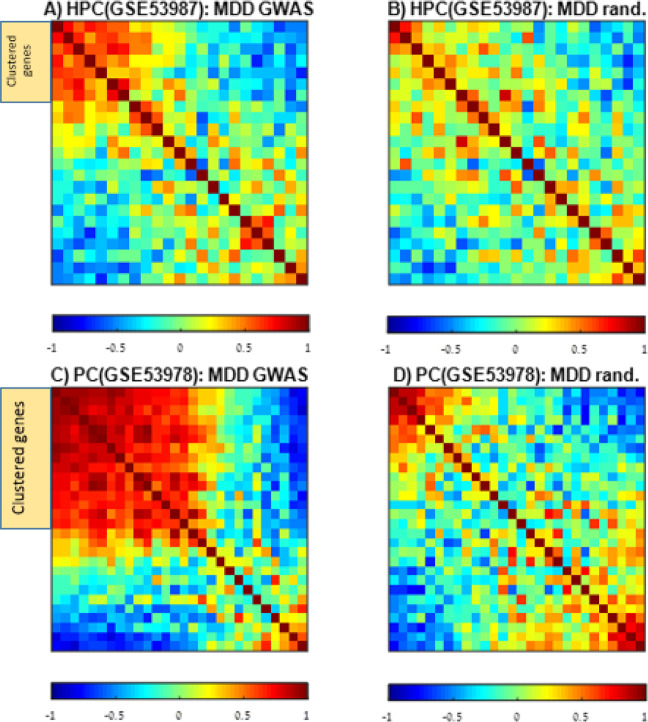
Correlation expression matrix of the GWAS-derived genes. The color in each entry (*x*,*y*) represents the correlation between the expression of genes *x* and *y*, along with all MDD samples in the specified brain region. Red color indicates high-correlated expression and blue, low. We used the SPIN tool [40]) to sort the list of genes so that genes with similar expression patterns would be grouped together. **A** The Pearson correlation expression matrix of the 23 GWAS-derived genes was calculated along with hippocampal (HPC) samples of MDD patients (GSE53987 data). Note that 23 of the 37 GWAS-derived genes were present in GSE53987. The orange rectangle represents the cluster of genes with highly correlated expression. **B** The Pearson correlation expression matrix of 23 randomly selected genes was calculated along with HPC samples of persons with MDD (GSE53987 data). **C** The Pearson correlation expression matrix of the 28 GWAS-derived genes calculated along parietal cortex (PC) samples of MDD patients (GSE53978 data). Note that 28 of the 37 GWAS-derived genes were present in GSE53978. The orange rectangle represents the cluster of genes with highly correlated expression. **D** The Pearson correlation expression matrix of 28 randomly selected genes was calculated along with PC samples of persons with MDD (GSE53978 data).

We analyzed the expression pairwise correlation patterns of the GWAS-based genes using the GSE53987 dataset (which includes 23 of the 37 genes), composed of hippocampal (HPC;16 samples), striatal (STR;15 samples), and Brodmann area (BA) 46 (17 samples) of individuals with MDD. The results for the HPC GSE53987 MDD samples (Fig. 1A) show a gene cluster with highly correlated expression (marked by an orange rectangle). We estimated the statistical significance of the correlation pattern in each of the three brain regions by generating 1000 random groups of genes of the same size (Supplementary Methods section 3). A typical correlation matrix for such a random group is shown in Fig. 1B. For the HPC (GSE53987) and STR (GSE53987), the correlation patterns showed high statistical significance: *p*-values of 0.006 and 0.02, respectively, for a threshold value of 5% (Fig. S2; Correlation matrices are presented in Fig. S3). The small *p*-values suggest a possible biological basis for the observed correlations and involvement in common biological pathways. We repeated this analysis for a more permissively defined GWAS-based gene list of 69 genes (Supplementary Methods section 1 and Fig. S4). The observation that this list did not yield statistically significant correlation patterns in the three brain regions (Fig. S2) suggests it contains false positives, i.e. genes that are not biologically associated with MDD.

### Searching for biological pathways in which MDD-associated genes play a significant role

We applied GeneAnalytics [18] PEA to the 37 GWAS-based genes (see Supplementary Methods section 5). Twenty superpathways were found to be enriched, with statistical significance. However, most of the superpathways contained only one of the 37 GWAS-based genes (See Table S3). These results are weak, as omitting a single gene from the GWAS-based gene group (for example, as a result of changing the upstream-downstream window around the peak SNP of each locus) eliminates enrichment of the superpathway that contains it.

To further verify the results, PEA was also applied using the Database for Annotation, Visualization, and Integrated Discovery (DAVID) tool [21] (see Supplementary Methods section 6). No statistically significant enriched pathways were detected using this method (score > 2), further supporting that the GeneAnalytics results are not reliable.

To improve pathway detection in MDD, based on GWAS-derived genes, we integrated gene expression analysis in the following way. For each of the three brain regions measured in GSE53987, we calculated a correlation matrix (see Supplementary Methods section 2) for the GWAS-based genes, using 23 of the 37 genes that are present in that dataset. Then, a cluster of highly correlated genes was selected for each brain region (for the HPC GSE53987, a cluster of 7 genes was identified: DENND1B, C6orf168, SLC30A9, DCC, DLST, RAB27B, and NEGR1; see the orange rectangle in Fig. 1A). For the STR GSE53987 and BA46 GSE53987, clusters of 8 and 6 genes, respectively, were identified (see Fig. S4). We focused on the HPC GSE53987, as its correlation pattern showed the most statistically significant results (see Supplementary Methods section 3 and Fig. S2B, D, and F). However, we note that the clusters of highly correlated genes identified in the STR and BA46 of GSE53987 significantly overlap with those of the HPC GSE53987 (7 of the 8 STR cluster genes; hypergeometric *p*-value = 0 and 3 of the 6 BA46 cluster genes; hypergeometric *p*-value = 0.045).

As demonstrated above, the correlation pattern of 23 of the 37 GWAS-based genes present in the HPC (GSE53987) was statistically significant. This suggests a possible biological basis for the observed correlations and involvement in common biological pathways. However, to enable statistically meaningful PEA of the GWAS-based genes, we expanded the list of genes. Following the rationale that genes that participate in a common biological pathway tend to have correlated expression [14], we expanded the cluster of HPC (GSE53987) highly correlated GWAS-based genes by an additional 793 genes whose expression levels are highly correlated with the cluster’s average profile (Pearson correlation ≥ 0.78, see Supplementary Methods section 4).

PEA was applied to the expanded list of genes using GeneAnalytics [18]. Table 2 presents selected results (full results of the 120 enriched pathways are presented in Table S4). While 800 is a somewhat arbitrary number of genes, the results were similar when the dataset was extended instead by 600 genes, with expression levels highly correlated with the cluster’s average profile (See Tables S5 and S6).

**Table 2:** Pathway enrichment analysis results of interest for 800 extended GWAS-based genes of the hippocampus (HPC) (GSE53987).

#	Enrichment score	Superpathway	# Matched (Superpathway) genes	PC (GSE35978) Enrichment score	DAVID corresponding pathway
1	48.48	Vesicle-mediated transport	60 (659)	25.98	Vesicle
2	40.04	Innate immune system	117 (2128)	16.14	–
3	38.39	Synaptic vesicle cycle	20 (90)	31.85	Synaptic vesicle
4	27.99	Ubiquitin-proteasome dependent proteolysis	19 (123)	12.27	Proteasomal ubiquitin-dependent protein catabolic process
5	27.92	Phagosome	21 (152)	–	–
6	27.80	tp53 regulates metabolic genes	16 (85)	–	–
7	26.02	Mitotic metaphase and anaphase	22 (180)	14.47	Anaphase-promoting complex-dependent proteasomal ubiquitin-dependent protein catabolic process
8	21.26	GABAergic synapse	21 (201)	30.49	–
9	15.88	Neuroscience	25 (341)	30.61	–
10	15.13	Cytokine signaling in immune system	42 (760)	–	–
11	14.49	MTOR Pathway	12 (103)	19.14	–
12	13.17	TGF-beta pathway	36 (653)	10.13	–
13	13.00	MTOR signaling pathway (KEGG)	17 (211)	11.9	–
14	12.25	Long-term potentiation	11 (104)	23.50	–
15	10.15	Brain-derived neurotrophic factor (BDNF) signaling pathway	12 (144)	18.88	–

Enrichment score (second column) corresponds to the log2-transformation of the *p*-value, corrected for multiple comparisons. High (green): corrected *p*-value ≤ 0.0001; medium (orange): 0.0001 < corrected *p*-value ≤ 0.05. The number (#) of matched genes (fourth column) corresponds to the number of genes in the 800 extended list that are included in each of the superpathways. The number of genes composing each of the superpathways appears in brackets. The PC (GSE35978) enrichment score (fifth column) corresponds to the same measure as in the second column, when measured using the PC (GSE35978) gene expression data. The DAVID^21^ corresponding pathway (sixth column) contains the most similar pathway found in the DAVID tool results.

Comparing the results to those of the GWAS-based genes alone (listed in Table S3), the extension of the list of implicated genes increased the statistical significance of the results and the number of enriched superpathways identified.

To further test the strength of the enriched pathways resulting in GeneAnalytics [18], we also applied the DAVID [21] PEA website tool and compared the results (See Table S7). The size of the overlap between the two independent PEA tools corresponds to an estimated hypergeometric *p*-value of 2.19 × 10^–11^ (see Supplementary Methods section 6 and 7). See the last columns of Table 2 and Table S4 for the GeneAnalytics superpathways that overlap DAVID resulting pathways.

### Replication in an independent dataset

To further validate our results, we repeated the analysis using independent gene expression data of post-mortem brain samples (GEO accession number GSE35978), which contains samples from the parietal cortex (PC) and the cerebellum (CRBLM). Note that 28 of the 37 GWAS-based genes are expressed in this dataset. See Fig. 1C for the correlation expression matrix of the PC GSE35978 MDD samples and Fig. 1D for a typical correlation matrix of a random group of genes.

We calculated the statistical significance of the correlation pattern of the two brain regions of GSE35978 (Fig. S2H, J). While for the CRLBM, a statistically significant correlation pattern was not observed, the correlation pattern of the PC was statistically significant (*p*-value of 0.006 for a threshold value of 5%). We thus decided to further explore the replicability of the results using the PC data.

A cluster of 16 highly correlated genes was identified for PC GSE35978 (Fig. 1C, marked by an orange rectangle): SLC30A9, BAG5, NEGR1, RSRC1, DENND1B, DLST, TMEM106B, L3MBTL2, SORCS3, RAB27B, TCF4, RBFOX1, LINC00461, LRFN5, TENM2, and DCC. Examining HPC GSE53987 (7 genes) and PC GSE35978 (16 genes) clusters, 6 of the 7 HPC genes are present in the PC cluster. Assuming that the clusters of highly correlated genes are random and uniformly distributed along the GWAS-based genes for both the HPC and PC, the size of their overlap corresponds to a hypergeometric *p*-value = 0. This suggests that these positive correlations are unlikely to be false positives, and plausibly represent involvement in common biological pathways.

Of the 17 unique genes with highly correlated expression in the HPC and/or PC, 11 were previously associated with MDD (Table S8). This provides further validation for their potential involvement in this disease.

The same correlation pattern analysis was applied to the healthy control samples (Fig. S5). While for region HPC (GSE53987) a statistically significant correlation pattern was not observed, for PC (GSE35978) the *p*-value was 0.002, for a threshold value of 5%. Out of 7 genes in the HPC (GSE53987) healthy controls cluster, 6 appeared in the HPC MDD cluster, corresponding to a hypergeometric *p*-value of 4.08 × 10^–6^. Of 15 genes in the PC (GSE35978) healthy controls cluster, 12 appeared in the PC MDD cluster (hypergeometric *p*-value = 0.001).

We note the statistically significant correlation pattern in the samples of the PC healthy controls (GSE35978), and the high replicability between the MDD and the controls, of the genes, composing the highly correlated clusters of both PC (GSE35978) and HPC (GSE53987). These observations suggest the involvement of the GWAS-based genes in common biological pathways that are not specific to the MDD samples. This infers involvement of GWAS-based genes in general biological processes, in both the MDD and the control samples.

**Fig. 2:**
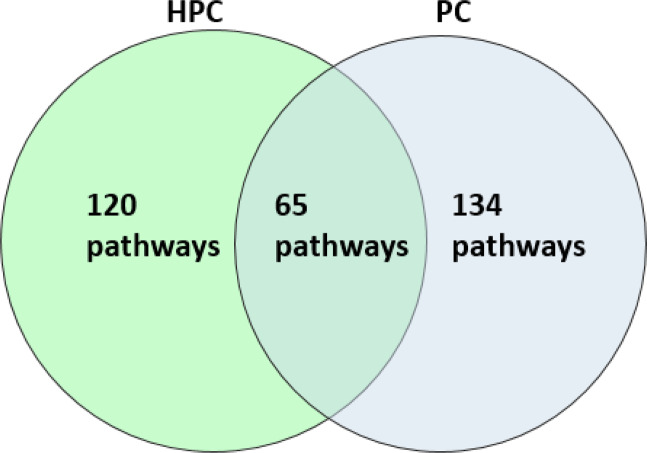
The overlap in the pathways enriched by GeneAnalytics [18], between the GWAS-based extended lists of the hippocampal (HPC) (GSE53987) and parietal cortex (PC) (GSE35978) datasets. For the respective datasets, 120 and 134 pathways were found to be enriched by GeneAnalytics. The size of the overlap between the resulting enriched pathways of the two datasets, 65, corresponds to a hypergeometric *p* value of 1.69 × 10^−^^34^.

We next expanded the GWAS-based genes cluster of PC (GSE35978) into a larger group of 800 genes whose expression levels are highly correlated with the cluster’s average profile (Pearson correlation ≥ 0.92). PEA was applied to the expanded group of genes; results are listed in Table S9 for GeneAnalytics and Table S10 for DAVID. As shown in Fig. 2, 65 of the 120 HPC-based (GSE53987) enriched pathways were replicated in the 134 PC-based (GSE35978) enriched pathways. The size of the overlap between the two independent datasets corresponds to a hypergeometric *p*-value of 1.69 × 10^−34^.

### Pathway-based differential expression analysis

Gene-level differential expression analysis of both the GWAS-based genes and the genes of the pathways that were found to be enriched did not yield statistically significant results (see Supplementary methods section 9, Tables S11 and S12). However, this is concordant with the accepted notion of the small effect of each of the numerous genes involved in the pathogenesis of MDD. Nonetheless, investigating the genes as a group might expose subtle changes in expression, which may collectively have an impact on relevant biological pathways. In addition, in the case that pathway-based differential expression is replicated between different brain regions and between independent datasets, this would significantly increase the validity of the results. We thus decided to explore pathway differential expression and compare the results between the various datasets and regions examined.

We first explored whether the gene expression datasets of the different brain regions ([STR, HPC, BA46—GSE53987], [PC, CRBLM—GSE35978]) are comparable. For each pair of brain regions, we applied a global correlation measure to estimate their comparability (see Methods section 6 and Table S13 for the correlation matrix). Examining the matrix shows significant positive correlations (defined as rho > 0.1 and *p*-value < 0.001) only between regions that originate from the same dataset (GSE53987). Thus, we conclude that the two datasets (GSE53987 and GSE35978) are not comparable in terms of differential gene expression.

To search for robust patterns, which appear in at least two independent datasets, we analyzed three additional datasets: GSE92538 (DLPFC data), Stanley Study ID 6 (Cerebellum data, by Allen A. Fienberg), and Stanley Study ID 16 (Thalamus data) from the Stanley Medical Research Institute (https://www.stanleygenomics.org/). We repeated the correlation analysis for the group of 5 (2 + 3 additional) datasets (Table S13). Brain regions STR (GSE53987), HPC (GSE53987), and DLPFC (GSE92538), originating from two independent datasets, were shown to be significantly positively correlated with each other (STR-HPC rho = 0.14, *p*-value = 2.39 × 10^–91^, HPC-DLPFC rho = 0.25, *p*-value = 3.6 × 10^–139^, STR-DLPFC rho = 0.14, *p*-value = 3.38 × 10^–45^). We concluded that these three brain regions are comparable in terms of differential expression.

### Proteasome-related genes are upregulated in human MDD brain and blood samples

**Fig. 3:**
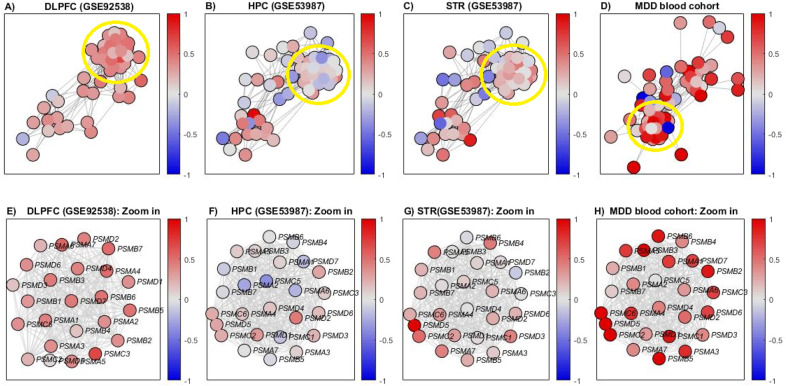
Pathway-based differential expression analysis of the ubiquitin-proteasome dependent proteolysis superpathway. **A** Differential expression of the ubiquitin-proteasome-dependent proteolysis superpathway measured on dorsolateral prefrontal cortex (DLPFC) (GSE92538) data. The colors of the nodes represent the deviation in expression of the MDD samples from the healthy control group; the edges represent relations of co-expression of the STRING database (see Supplementary Methods section 7). Only genes with relation to at least one other gene in the superpathway are displayed. The yellow circle represents a subgroup of highly interconnected proteasome subunit genes. **B** The same as in A) for the hippocampal (HPC) (GSE53987) data. **C** The same as in A) for the striatal (STR) (GSE53987) data. **D** The same as in (**A**) for the MDD PBMC cohort. **E** Zoom-in on the proteasome subunit genes that are circled in (**A**). **F** Zoom-in on the proteasome subunit genes that are circled in (**B**). **G** Zoom-in on the proteasome subunit genes that are circled in (**C**). **H** Zoom-in on the proteasome subunit genes that are circled in (**D**). Note that there is a blue-colored gene in (**D**) which is circled, UBE2V2, which does not appear in (**H**) as it does not encode a proteasome subunit.

Examining the enriched pathways of interest presented in Table 2 reveals immune-system pathways (for example, the innate immune system and TGF beta pathway), which are known to be involved in MDD [22,23,]. Several brain-related biological processes (for example, synaptic vesicle cycle and long-term potentiation) were also found to be enriched (Table 2). While the inclusion of these pathways among the resulting enriched pathways increases the validity of our results, we decided to focus on the ubiquitin-proteasome dependent proteolysis pathway, for the following reasons: (i) Its enrichment, which was originally found in the HPC (GSE53987), was replicated in an independent dataset, PC (GSE35978), and when using both GeneAnalytics [18] and DAVID [21] PEA tools (Table 2). (ii) When applying pathway-based differential expression analysis (see Supplementary Methods section 7), the pathway showed a tendency for upregulation in all three brain regions: STR (GSE53987), HPC (GSE53987), and DLPFC (GSE92538) (Fig. 3). Binomial *p*-values for this tendency (Supplementary Methods section 8) were significant: *p*-values = 0.32 × 10^–4^ for STR (GSE53987), 0.0041 for HPC (GSE53987), and 5.86 × 10^–10^ for DLPFC (GSE92538). (iii) While some evidence exists for its involvement in MDD [24,25,], and in additional psychiatric disorders such as schizophrenia, bipolar disorder, and autism spectrum disorder [26,27,], to our best knowledge the differential expression of this pathway has not been studied in MDD.

When examining Fig. 3, a subgroup of highly interconnected genes that encode for proteasome subunits is apparent. The proteasome subunits tend to be up-regulated in the three brain regions (Fig. 3E–G). Interestingly, when the same analysis was applied to a cohort of 9 MDD blood samples versus 9 controls, that were collected at Shalvata Mental Health Center and subjected to RNA-sequencing in PBMCs, the proteasome subunits showed a clear tendency for up-regulation (Fig. 3H). Again, when gene-level differential expression analysis was performed, no gene reached statistical significance after correction for multiple comparisons, although 4 proteasome subunits genes were found to be up-regulated in MDD PBMCs with *p*-value ≤ 5% (Table S14)

## Discussion

In this study, we integrated MDD GWAS results with gene expression data, with the aim of interpreting GWAS results with regard to the biological pathways involved. The integration of GWAS results with gene expression data poses several advantages: (1) Potential decreases in the false-positive rates that result when the data sources are considered separately. (2) GWAS results often identify loci that regulate gene expression rather than affect protein structure [28]. (3) Gene expression data also include effects of epigenetic changes. GWAS-based gene expression data showed significant pairwise correlations in three of the five brain regions measured: HPC, STR (GSE53987), and PC (GSE53987). This suggests the involvement of these genes in common biological processes. The integration with gene expression data enabled investigating additional genes whose expression patterns have been shown to correlate with the average profile of a cluster of highly correlated GWAS-based genes. This step follows the rationale that the expression patterns of genes involved in a given biological pathway tend to be correlated. We showed that expansion of the genes investigated enabled performing statistically meaningful PEA that was not possible when considering only GWAS-based genes. This methodology was previously applied to schizophrenia, for which it was also shown to improve the ability to perform PEA to GWAS-resulting genes [10,13,]. We replicated the results of the PEA, based on the integration of GWAS-based genes with gene expression data, using two independent datasets, HPC (GSE53987) and PC (GSE53978) (the overlap between the resulting enriched pathways corresponds to a hypergeometric *p*-value of 1.69 × 10^−34^).

While gene-level differential expression analysis did not yield statistically significant results, pathway-based differential expression analysis identified upregulation of the ubiquitin-proteasome dependent proteolysis pathway in three brain regions, measured in two independent datasets (GSE53987 and GSE92538) (Fig. 3). The resulting enriched pathways (Table 2) included immune-system pathways that are known to be involved in MDD and several brain-related biological processes, such as the synaptic vesicle cycle and long-term potentiation. However, we focused on the ubiquitin-proteasome-dependent proteolysis pathway, due to a number of reasons. (i) Its enrichment was replicated when using two independent gene expression datasets and when using two PEA tools. (ii) It showed a tendency for upregulation in the three brain regions tested (Fig. 3). (iii) While evidence exists for its involvement in MDD, to our best knowledge its differential expression has not yet been studied in MDD.

Our examination in greater depth of the pathway-based differential expression of the ubiquitin-proteasome-dependent proteolysis pathway (Fig. 3) led to the identification of a subgroup of highly interconnected genes that encode for proteasome subunits, with a tendency for up-regulation in MDD. This might indicate the involvement of these genes in the pathogenesis of MDD. The findings of several previous studies support this hypothesis. For example, in [24], the role of three proteasome subunit genes (PSMA7, PSMD9, and PSMD13) in the mechanisms underlying the resistance/response to antidepressants was explored, by genotyping 231 treatment-responsive and 390 treatment-resistant individuals with MDD. The PSMD13 rs3817629 G allele was found to be associated with treatment-resistant depression. In addition, individuals with homozygous GG of this SNP exhibited lower mRNA levels in fibroblasts for PSMD13 than did individuals with the A allele. Moreover, in another study [25], gene expression was measured by microarrays in blood samples of 34 patients with MDD and 33 matched controls. Using a machine learning algorithm, a 13-gene predictive model of response to antidepressants was constructed, with close to 80% accuracy. Interestingly, two of the 13 genes were proteasome subunit genes. Importantly, our analysis identified a clear tendency for up-regulation of proteasome subunits in MDD blood samples too, suggesting a potential role as biomarkers. However, as the analyzed cohort was relatively small (9 patients vs. 9 controls) this finding requires further validation.

The proteasome subunits have been found to be involved in additional psychiatric disorders. For example, global down-regulation of proteasome subunit genes was detected in multiple brain regions of post-mortem samples of 267 individuals with schizophrenia vs. 266 healthy controls in [26]. A PEA applied to differentially expressed genes of blood samples of 23 individuals with bipolar disorder and 24 healthy controls identified the ubiquitin-proteasome pathway as one of the top ten enriched pathways [29].

One hypothesis that arises from the above results is that the proteasome is involved in MDD through its role in immune system processes. This is supported by a number of propositions. (i) The proteasome is known to be tightly involved in various processes of the immune system, such as T cell repertoire selection, CD8 T cell responses, and antigen processing of MHC class I [30]. (ii) Several of the pathways that we found to be enriched in the extended list of GWAS-based genes are related to the immune system (Table 2), and closely involve proteasome subunit genes. Examples include cytokine signaling in the immune system [31], the innate immune system [32], and TGF-beta pathways [33]. The proteasome subunit genes are included in the list of genes that construct these pathways in GeneAnalytics. (iii) Gene expression of the proteasome subunits positively correlates with genes in the three-mentioned immune system-related pathways and also in the phagosome pathway (see Fig. S6). However, further study is needed to establish this hypothesis and to elucidate the involvement of the proteasome in immune system processes associated with MDD.

Another hypothesis that arises from the above results involves the signaling pathways that we found to be enriched in the extended list of GWAS-based genes, namely of the brain-derived neurotrophic factor (BDNF) and mammalian target of rapamycin (mTOR) (See Table 2). BDNF was shown to have elevated expression levels following antidepressant use [34] and to contribute to the regulation of mTOR signaling [35,36,]. mTOR signaling was shown, in turn, to be involved in the regulation of the ubiquitin-proteasome-dependent proteolysis pathway [37,38,]. To further explore this possible regulatory connection, we created a correlation matrix that compares the expression patterns of the genes that compose three pathways (BDNF, mTOR signaling, and the proteasome subunits) (Fig. S7). The high positive correlations between the genes composing these pathways support the suggested regulatory connection. However, additional study is needed to further establish the regulatory path that results in the upregulation of proteasome subunits in MDD.

A major limitation of post-mortem brain sample studies is the cellular complexity of the brain tissue and the diverse cell types that compose the brain samples. This can result in the dilution of authentic changes in gene expression in cell subpopulations, and yield false-negative reports. Indeed, differential expression analysis at the gene level did not reveal statistically significant differences. This is concordant also with the accepted notion that the pathogenesis of MDD involves tens or hundreds of genes, whereby the contribution of every single gene is small. Nonetheless, our application of pathway-based differential expression identified differential expression of the ubiquitin-proteasome-dependent proteolysis pathway. While the magnitude of the differential expression was mild, the replication in three brain regions from two independent datasets supports its validity.

Another important limitation of this study is the potential confounding effect of the use of antidepressants, which could alter gene expression levels [39]. This limitation was addressed by repeating the pathway-based differential expression analysis and comparing the findings of the individuals with MDD patients who were treated with antidepressants to those who were not. This information was available for one of the three datasets analyzed, GSE53987. Compared to those who did not take antidepressants (Fig. S8), those who took them did not show a greater magnitude of upregulation of the proteasome subunit genes. Thus, it is likely that antidepressant use is not responsible for the upregulation of the proteasome subunit genes that we detected.

As gene expression does not frequently correlate with the level of the proteins coded by the genes, drawing conclusions regarding the biological consequences of the signal we detected is impossible. However, our integration of GWAS results with gene expression data increases the validity of the association between the enriched pathways identified and the pathogenesis of MDD. Still, additional study is needed to further validate the up-regulation of the proteasome subunit genes that we detected, and to explore the consequences in terms of protein levels.

To summarize, by integrating GWAS results with gene expression data, we identified biological pathways that are associated with MDD. Pathway-based differential expression analysis detected up-regulation of proteasome subunit genes in MDD. While this signal was replicated in three brain regions: HPC, STR, and DLPFC and also in PBMCs, further validation of this signal and exploration of its impact at the protein level are required. We have shown that the integration of two data sources, GWAS (DNA studies) and RNA expression levels, improves the ability to interpret MDD GWAS results in terms of the relevant neurobiological pathways.

## Supplementary information

Supplementary Materials

Supplementary Tables
