# Supplementary material for: Genome wide analysis implicates upregulation of proteasome pathway in major depressive disorder
Source: Transl Psychiatry. 2021 Jul 28;11:409. doi: 10.1038/s41398-021-01529-x (PMC8319154; doi:10.1038/s41398-021-01529-x)
Supplement: Supplementary file 1 — Supplementary Materials [file 41398_2021_1529_MOESM1_ESM.docx]

**Genome wide analysis implicates up-regulation of proteasome pathway in major depressive disorder**

Shaked Balaish^1^, Ifat Israel-Elgali^1,2^, Guy Shapira^1,3^, Israel Krieger^1,4^, Aviv Segev^1,4^, Uri Nitzan^1,4^, Michael Majer^5^, Yuval Bloch^1,4^, Abraham Weizman^1,2,6^, David Gurwitz^1,2^, Noam Shomron^1,2,3*^, Libi Hertzberg^1,4,5*^

^1^ Sackler Faculty of Medicine, Tel Aviv University, Tel Aviv, Israel

^2^ Sagol School for Neuroscience, Tel Aviv University, Tel Aviv, Israel

^3^ Edmond J. Safra Center for Bioinformatics, Tel Aviv University, Tel Aviv, Israel

^4^ Shalvata Mental Health Center, Hod Hasharon, Israel

^5^ Department of Physics of Complex Systems, Weizmann Institute of Science, Rehovot, Israel

^6^ Geha Mental Health Center, and Felsenstein Medical Research Center ,Petah Tikva, Israel

* Correspondence could be sent to [libi.hertzberg@gmail.com](mailto:libi.hertzberg@gmail.com) or [nshomron@post.tau.ac.il](mailto:nshomron@post.tau.ac.il)

**Supplementary Methods:**

Table of Contents

[1. GWAS-based gene lists: 3](#_Toc52824838)

[2. Pre-processing of mRNA expression: 3](#_Toc52824839)

[3. Calculation of a p-value of the observed correlation pattern 4](#_Toc52824840)

[4. Quality control using the principal component analysis method 5](#_Toc52824841)

[5. Comparing GeneAnalytics results, using 800, and subsequently 600, highly correlated genes 5](#_Toc52824842)

[6. Pathway enrichment analysis using the DAVID - Functional Annotation Tool 5](#_Toc52824843)

[7. Comparing GeneAnalytics and DAVID tools 6](#_Toc52824844)

[8. Calculating the tendency of up-regulation or down-regulation of a biological pathway in MDD vs. control samples using a binomial p-value 6](#_Toc52824845)

[9. Differential gene expression analysis between individuals with MDD and healthy controls 6](#_Toc52824846)

[10. MDD blood samples cohort 7](#_Toc52824847)

[Figure S1: PCA results for HPC, STR (GSE53987) and CRBLM (GSE35978). 9](#_Toc52824848)

[Figure S2: The statistical significance of the observed expression pairwise correlation patterns for the GWAS-based gene groups 1 and 2, calculated for each region separately 10](#_Toc52824850)

[Figure S3: The pairwise Pearson correlation matrix for GWAS-based gene group 1 and a random group of genes. 11](#_Toc52824851)

[Figure S4: The pairwise Pearson correlation matrix of GWAS-based gene groups 1 and 2 12](#_Toc52824852)

[Figure S5: GWAS-based gene group 1 pairwise Pearson correlation matrices and p-value calculation of the observed correlation patterns for healthy control samples of HPC (GSE53987) and PC (GSE35978), calculated for each region separately. 13](#_Toc52824853)

[Figure S6: The pairwise Pearson correlation matrix of the proteasome subunits and immune system-related pathways genes 14](#_Toc52824854)

[Figure S7: The pairwise Pearson correlation matrix of the proteasome subunits and mTOR+BDNF-related pathway genes 15](#_Toc52824855)

Figure S8: Pathway-based differential expression analysis of the proteasome subunits genes, comparing MDD patients who took antidepressants with those who did not, measured on GSE53987............................................................................................................................................................16

[Table S1: Expanded description of gene expression data. 17](#_Toc52824856)

Table S2: MDD blood samples cohort. [. 18](#_Toc52824857)

[Table S3: GeneAnalytics pathway enrichment results of the 37 GWAS-based gene group. 18](#_Toc52824858)

Table S4: GeneAnalytics pathway enrichment results of the 800 extended GWAS-based gene group from the HPC (GSE53987) dataset. - See supplementary tables .……………………………………….……………....19

[Table S5: Comparison between the resulting GeneAnalytics enriched pathways of the 800 and 600 genes that extended the GWAS-based gene group. The expression levels of these added genes are highly correlated with the average profile of brain regions PC (GSE35978) and HPC (GSE53987) of the GWAS-based gene cluster…………………………………………………………………………………………………………...19](#_Toc52824859)

[Table S6: Comparison between the highly scored GeneAnalytics enriched pathways that resulted from extending the gene groups by 800 and 600 genes. The expression levels of these added genes are highly correlated with the average profile of brain regions PC (GSE35978) and HPC (GSE53987) of the GWAS-based gene cluster 19](#_Toc52824860)

[Table S7: DAVID pathway enrichment results of the 800 extended GWAS-based gene group from the HPC (GSE53987) dataset. - See supplementary tables. 20](#_Toc52824867)

[Table S8: Supporting evidence for the involvement of the GWAS-based genes in MDD 20](#_Toc52824861)

[Table S9: GeneAnalytics pathway enrichment results of the 800 extended GWAS-based gene group from the PC (GSE35978) dataset. - See supplementary tables 20](#_Toc52824862)

[Table S10: DAVID pathway enrichment results of the 800 extended GWAS-based gene group from the PC (GSE35978) dataset. - See supplementary tables. 20](#_Toc52824868)

[Table S11: Differential expression analysis (two-sided t-test) results for each of the 23 GWAS-based genes of group 1 that are present in GSE53987, comparing HPC MDD samples and controls 21](#_Toc52824863)

[Table S12: Differential expression analysis (two-sided t-test) results for each of the 28 GWAS-based genes of group 1 that are present in GSE35978, comparing PC MDD samples and controls 22](#_Toc52824864)

[Table S13: Pairwise dataset comparability measures for three MDD gene expression datasets, applied to each region separately. 23](#_Toc52824865)

[Table S14: Top 10 differentially expressed proteasome subunits genes between PBMCs of 9 MDD patients and 9 healthy controls.. 24](#_Toc52824866)

[References 25](#_Toc52824869)

1. GWAS-based gene lists: Results were obtained from the latest MDD GWAS study, which applied a meta-analysis to 135,458 cases and 344,901 controls from 7 cohorts (Wray et al., 2018). That study identified 44 independent, statistically significant loci as associated with MDD. We listed the genes located 200 kb downstream and upstream to each of the 44 associated loci (Wray et al., 2018). Since the risk loci often span large genomic regions, a major challenge is to identify the gene(s) relevant to these loci. To tackle this challenge, we created two sorts of GWAS-based gene lists, of different permissiveness criteria: (i) The closest gene (within a window of 200 kb downstream and upstream) to the peak SNP of each of the 44 resulting associated loci. This list contains 37 genes in total, 23 of which exist in the [GSE53987](https://www.ncbi.nlm.nih.gov/gds/?term=GSE53987%5bAccession%5d) gene expression dataset. (ii) All the genes within a window of 200 kb, downstream and upstream to the 44 resulting associated loci. This list contains 69 genes, 37 of which exist in the [GSE53987](https://www.ncbi.nlm.nih.gov/gds/?term=GSE53987%5bAccession%5d) gene expression dataset. This list is expected to include a relatively high rate of false positives (i.e. genes that are not biologically associated with MDD).

Notably, the size of the window is somewhat arbitrary, usually ranging from 5 to 500 kb (Petersen et al., 2013), according to the literature. We used 200 kb, as suggested in the latest MDD GWAS (Wray et al., 2018), and as has been used in other studies (Kampe et al., 2019; Le et al., 2019; Zhang et al., 2016)

### 2. Pre-processing of mRNA expression:

[GSE53987](https://www.ncbi.nlm.nih.gov/gds/?term=GSE53987%5bAccession%5d):

We preprocessed the microarrays’ raw data, given as CEL files, using the HG-U133_Plus_2 cdf file, by running the MATLAB function affyrma (CELFiles, CDFFile). This function performs Robust Multi-array Average (RMA) normalization to the Affymetrix microarray probe-level data. It outputs log2 based gene expression values that have been background adjusted, normalized, and summarized (Irizarry et al., 2003).

At this point we have 54,675 probe sets. We converted the probe sets to gene symbols using the annotation file from the Affymetrix webpage, and removed probe sets that did not have a corresponding gene symbol.

(<http://www.affymetrix.com/support/technical/byproduct.affx?product=hg-u133-plus>).

For genes that were represented by more than one probe-set, we kept the probe-set with the highest mean expression level measured along all the samples. After this filtering, 20,866 genes remained.

GSE35978:

We downloaded the Series Matrix file, which contains RMA-normalized expression values from the sample data tables. At this point we have 25,293 probe sets. We converted the probe sets into gene symbols using the annotation file from the Affymetrix webpage, and removed probe sets that did not have a corresponding gene symbol.

(<http://www.affymetrix.com/support/technical/byproduct.affx?product=hugene-1_0-st-v1>).

For genes that were represented by more than one probe-set we kept the probe-set with the highest mean expression level through all the samples. After this filtering, 23,307 genes remained.

GSE92538:

We downloaded the Series Matrix file, which contains RMA-normalized expression values from the sample data tables. At this point we have 11,973 probe sets. We converted the probe sets into gene symbols using the annotation file from the Affymetrix webpage and removed probe sets that did not have a corresponding gene symbol.

(<http://www.affymetrix.com/support/technical/byproduct.affx?product=hg-u133-plus>).

For genes that were represented by more than one probe-set we kept the probe-set with the highest mean expression level considering all the samples. After this filtering, 11,865 genes remained.

### 3. Calculation of a p-value of the observed correlation pattern

Given the pairwise Pearson correlation coefficients that were calculated in section 3 of the GWAS-based gene group, we estimated the probability of obtaining by chance, the same or higher absolute correlation values. We examined the absolute values in order to consider both low negative-correlation values and high positive-correlation values. We were interested in estimating the probability of obtaining at least the number of "highly correlated" pairs of genes that was calculated in a random group with the same number of genes.
We calculated the pairwise correlation values of 1000 groups of the same size as the given GWAS-derived gene group. These were randomly selected from the whole dataset of genes. Accordingly, we generated a distribution of the absolute values of the pairwise correlations. We then estimated the probability of obtaining by chance at least the number of "highly correlated" pairs of genes, as of the GWAS-derived gene group.

We sought to define the correlation values that are considered “high”. Thus, we used the p-values calculated for each of the pairwise Pearson correlation coefficients. These p values reflect the probability of obtaining such a correlation value, or higher, when the two vectors containing the expression levels of each pair of genes are randomly permutated. We repeated this for different cutoffs of the p-value for the Pearson correlation coefficients (5%, 1%, 0.1%, 0.01% and 0.001%); these values were used as thresholds. For each p-value threshold, we calculated the fraction of the 1000 random groups of genes, for which the number of gene pairs with Pearson correlation p-values below the threshold is higher than in the given GWAS-derived gene group.

### 4. Quality control using the principal component analysis method

Principal component analysis (PCA) is a statistical tool for reducing the dimensions or variants in multivariate data, while increasing interpretability and minimizing information loss. Instead of investigating hundreds and thousands of original variants, the first few variants contain the majority of the data's variation and can be investigated solely. In our case, to identify outlier samples, we investigated the first two variants of the data, while examining the 3000 genes with the highest standard deviation. This was applied to each of the brain regions separately (STR, HPC, BA46 [GSE53987], PC, CRBLM [GSE35978] and DLPFC [GSE92538]). In HPC, STR and CRBLM we found three samples overall whose expression patterns differed greatly from those of the other samples, as can be seen in figure S1 (sample GSM1304898 from the HPC, sample GSM1305037 from the STR, and sample GSM878286 from the CRBLM data). Following these results, we excluded these three samples from the remaining analyses.

### 5. Comparing GeneAnalytics results, using 800, and subsequently 600, highly correlated genes

To test the robustness of the results of the pathway enrichment analysis (PEA), we compared GeneAnalytics results, using the 800, and subsequently the 600 genes with the highest pairwise correlation to the cluster’s average profile. The results were compared separately for each brain region. The results were compared twice, for all the resulting enriched pathways, and for the highly scored enriched pathways (highly scored pathways are defined by GeneAnalytics as those with corrected p-values smaller or equal to 0.0001). Tables S1 and S2 show a statistically significant overlap between the results, with very low p-values (highest p-value = 1.33×10^-57^). This provides a measure for the robustness of the resulting enriched pathways, which did not change dramatically when the number of input genes was slightly changed.

### 6. Pathway enrichment analysis using the DAVID - Functional Annotation Tool

Given a group of genes, the Database for Annotation, Visualization and Integrated Discovery (DAVID) tool was applied to identify enrichment of biological pathways. We chose to display DAVID results similarly to GeneAnalytics superpathways. Thus, we downloaded DAVID results as a Functional Annotation Clustering Tool, which is designed to cluster similar terms based on the overlap of genes in a given list. The DAVID enrichment score is the geometric mean (in –$\log_{10}$scale) of all a pathway’s p-values in a corresponding annotation cluster. The rule of thumb is to focus on enrichment scores >=2 (p-value=<0.01).

### 7. Comparing GeneAnalytics and DAVID tools

To test the robustness of our results, we compared the results of GeneAnalytics and DAVID tools, for both HPC ([GSE53987](https://www.ncbi.nlm.nih.gov/gds/?term=GSE53987%5bAccession%5d)) and PC ([GSE53978](https://www.ncbi.nlm.nih.gov/gds/?term=GSE53987%5bAccession%5d)) regions, while applying (PEA) to the extended list of GWAS-based genes.

For HPC ([GSE53987](https://www.ncbi.nlm.nih.gov/gds/?term=GSE53987%5bAccession%5d)), 32 of 85 high-ranked (p-value < 0.0001) superpathways from GeneAnalytics, and 15 of 29 significant (p=value<0.01) clustered-pathways from DAVID, constituted identical or similar pathways in the alternative tool. The size of the overlap between the two independent PEA tools corresponds to an estimated hyper-geometric p-value of 2.19×10^-11^. See Table S11 for GeneAnalytics results; the superpathways that overlap with the DAVID results appear in the last column.

For PC ([GSE53978](https://www.ncbi.nlm.nih.gov/gds/?term=GSE53987%5bAccession%5d)), 16 of 76 high-ranked (p-value < 0.0001) pathways from GeneAnalytics and 10 of 23 significant (p=value<0.01) pathways from the DAVID tool, had an identical or similar pathway name in the alternative PEA tool. The size of the overlap corresponds to an estimated hyper-geometric p-value of 8.65×10^-8^. See Table S12 for GeneAnalytics results; the superpathways that overlap with the DAVID results appear in the last column.

### 8. Calculating the tendency of up-regulation or down-regulation of a biological pathway in MDD vs. control samples using a binomial p-value

To examine whether a biological pathway has a tendency for up-regulation or down-regulation in MDD, we applied a binomial p-value calculation for the number of genes in the pathway with (even slightly) increased (or decreased) expression in MDD vs. the control group. The MATLAB function “bnocdf” was used. This computes a binomial cumulative distribution function as follows:

[y = binocdf(x,n,p)](https://www.mathworks.com/help/stats/binocdf.html#d117e140894)

x = for each gene we calculated whether its mean expression is higher or lower in the MDD samples vs. the controls, and counted the number of genes in the given pathway that are (even slightly) up- or down-regulated.

n = the number of genes in a specific pathway (number of trials).

p = 0.5 (probability of “success” (up- or down-regulation) for each trial, given a random group of genes)

A statistically significant p-value indicates that the given pathway has a tendency for up- or down-regulation, which is unlikely when assuming that the input genes are a random group.

### 9. Differential gene expression analysis between individuals with MDD and healthy controls

The two-sided t-test was conducted for HPC (GSE53987) and PC (GSE35978) regions separately, to examine differences in mRNA expression levels between individuals with MDD and healthy controls.

We first applied the two-sided t-test to the original GWAS-based genes (list #2) for HPC GSE53987 (23 genes) and PC GSE35978 (28 genes) regions separately. For two genes, LINC00461 and DLST, the p-values were < 0.05 (0.0319 and 0.0127, respectively) when compared between PC GSE35978 MDD and controls. However, after applying the Benjamini and Hochberg false discovery rate (FDR) method for multiple comparison correction (Benjamini and Hochberg, 1995), none of the genes showed statistically significant differential expression in MDD versus control samples (FDR<0.05). For the full results see Tables S6 and S7.

We then applied the two-sided t-test to the GeneAnalytics resulting enriched pathways. Only genes from our extended gene list (800 genes) were included. For example, the pathway “Metabolism”, which has the highest score of the HPC GSE53987 enriched pathways, is composed of 2577 genes in total, 162 of them from our extended gene list. In this case, we conducted a two-sided t-test on the mRNA expression levels of the 162 genes, between MDD and controls, for each of the HPC GSE53987 and PC GSE35978 regions separately. Differential expression was not statistically significant for any of the genes, comparing MDD versus control samples, for both groups, before and after the FDR correction (p-value <0.05 and FDR<0.05).

### 10. MDD blood samples cohort

**Blood separation**

Blood samples were collected at Shalvata Mental Health Center in EDTA containing tubes (purple cap Vacutainer^TM^ tubes). Tubes were transferred to our lab and blood was separated using UNI-SEP Lymphocyte Separation Tubes (Novamed, Israel). Whole blood was transferred to UNI-SEP tubes (U-04 tubes by Novamed, Israel), and centrifuged for 15 minutes at 2700 RPM, to receive plasma and peripheral blood mononuclear cells (PBMCs). The layer containing PBMCs was transferred to a new 15 ml tube with approximately 7 ml PBS intended for washing, and centrifuged for 10 minutes at 1700 RPM. Supernatant was discarded and PBMCs were gently resuspended in 3 ml PBS and transferred to two 1.5 ml Eppendorf tubes. Tubes were centrifuged for 5 minutes at 1700 RPM. Supernatant was discarded and tubes with PBMCs were frozen. Tubes were stored at minus 80^o^C. For storage periods above 6 months, PBMC samples were stored in the vapor phase of a liquid nitrogen freezer (below -190^o^C).

**RNA extraction**

Samples underwent RNA purification using phenol-chloroform extraction: PBMC samples were lysed using TRIzol reagent (Thermo Fisher Scientific, USA), followed by RNA separation using chloroform and isopropanol precipitation. The final RNA concentration and purity were measured using a NanoDrop ND-1000 spectrophotometer (NanoDrop Technologies, Thermo Fisher Scientific, USA).

**RNA sequencing**

Total RNA samples were delivered on dry ice to Macrogen, South Korea, for sequencing.  Libraries were prepared using TruSeq Stranded Total RNA LT Sample Prep Kit (Globin). RNA sequencing was performed according to the manufacturer’s instructions (Illumina).

**Preprocessing**

Raw sequencing data was trimmed and using fastp 0.19.6 (Chen et al. 2018) and filtered using RNA-QC-Chain, then aligned to the GRCh38 assembly and using STAR 2.7.1a (Dobin et al. 2013). Batch correction was performed using SVA 3.36.0 (Leek et al. 2012).

**Differential expression analysis**

Differential gene expression was applied using DESeq2, which was shown to have high sensitivity and precision when applied to RNA-seq data (Love, Huber, and Anders 2014). The expression of each of the 54 proteasome subunits encoding genes, that were expressed in the MDD blood samples cohort, was compared between the 9 MDD patients and the 9 controls, with age and gender as additional covariates. See the results for the 10 top differentially expressed proteasome subunits genes in Table S10. Correction for multiple comparisons was performed using the Benjamini and Hochberg false discovery rate (FDR) method (Benjamini and Hochberg, 1995).

**Figures**


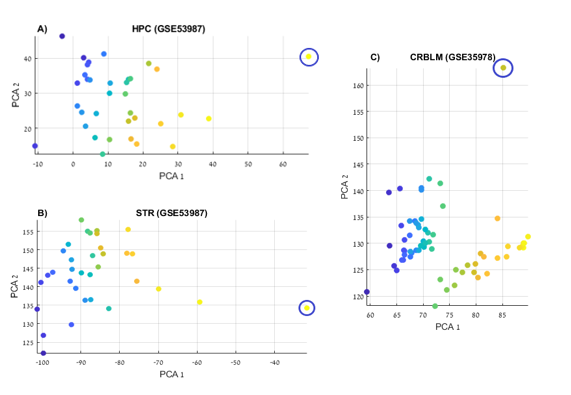


Figure S1: PCA results for HPC, STR (GSE53987) and CRBLM (GSE35978). Principal component analysis (PCA) is a statistical method that can reduce the number of the original variables. Here we examined the two variables with the greatest difference in their values, and noticed 3 samples that are considerably different from the remaining samples. Following these results, we excluded these 3 samples from our datasets. A) PCA applied for the HPC samples (MDD and controls) of GSE53987. One outlier (GSM1304898) is marked. B) PCA applied for the STR samples (MDD and controls) of GSE53987. One outlier (GSM1305037) is marked. C) PCA applied for the CRBLM samples (MDD and controls) of GSE35978. One outlier (GSM878286) is marked.


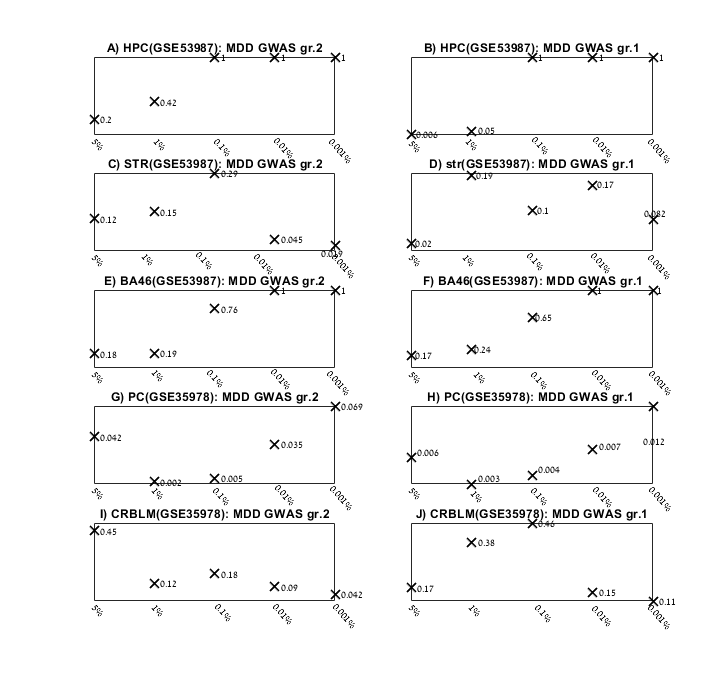


Figure S2: The statistical significance of the observed expression pairwise correlation patterns for the GWAS-based gene groups 1 and 2 (more permissive), calculated for each region separately. The x-axis label represents the P-value thresholds of the pairwise correlation values. The y-axis represents the p-value of the observed pairwise correlation values of the given GWAS-derived gene group, calculated as described in Supplementary methods section 3. Each “x-number” represents the calculated p-value that corresponds to the threshold represented by the x-axis.

Figure S3: The pairwise Pearson correlation matrix for GWAS-based gene group 1 and a random group of genes. Pairwise Pearson correlation matrices were calculated for GSE53987 and GSE35978 datasets using GWAS-based gene group 1, and compared to a random group of genes of the same size, for each region separately. Each entry (i,j) represents the Pearson correlation coefficient between the expression of genes i and j along the MDD samples. The genes are sorted by the SPIN tool (Tsafrir et al., 2005). For a random group of genes, a smaller number of pairwise highly correlated genes was observed. A) The pairwise Pearson correlation matrix measured on the STR MDD samples of GSE53987 for the GWAS-based gene group 1. B) The same as in A), for a random group of genes of the same size. C) The pairwise Pearson correlation matrix measured on the BA46 MDD samples of GSE53987 for the GWAS-based gene group 1. D) The same as in C), for a random group of genes of the same size. E) The pairwise Pearson correlation matrix measured on the CRBLM MDD samples of GSE35978 for the GWAS-based gene group 1. F) The same as in E), for a random group of genes of the same size.

Clustered genes

Clustered genes

Figure S4: The pairwise Pearson correlation matrix of GWAS-based gene groups 1 and 2 (more permissive). Pairwise Pearson correlation coefficients measured on the MDD samples of the GSE53987 dataset, for the 2 GWAS-based gene groups (see Methods section 1.2) are plotted in a matrix, for each region separately. Each entry (i,j) represents the Pearson correlation coefficient between the expression of genes i and j along the MDD samples. The genes are sorted by the SPIN tool (Tsafrir et al., 2005). GWAS-based group 2 is defined as all genes within a distance of 200 kb downstream and upstream from the 44 GWAS-resulting associated loci. This group contains 69 genes, 37 of them exist in the GSE53987 dataset. GWAS-based group 1 is defined as the closest gene to the peak SNP of each of the 44 GWAS-resulting associated loci. This group contains 37 genes in total, 23 of them exist in the GSE53987 dataset. A) The pairwise Pearson correlation matrix measured on the HPC MDD samples of GSE53987 for the GWAS-based gene group 2. B) The same as in A), for the GWAS-based gene group 1. C) The pairwise Pearson correlation matrix measured on the STR MDD samples of GSE53987 for GWAS-based gene group 2. D) The same as in C), for the GWAS-based gene group 1. E) The pairwise Pearson correlation matrix measured on the BA46 MDD samples of GSE53987 for the GWAS-based gene group 2. F) The same as in E), for the GWAS-based gene group 1.

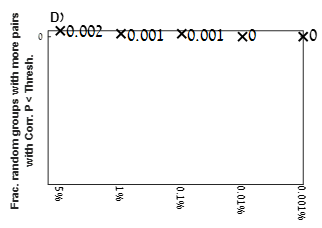


Figure S5: GWAS-based gene group 1 pairwise Pearson correlation matrices and p-value calculation of the observed correlation patterns for healthy control samples of HPC (GSE53987) and PC (GSE35978), calculated for each region separately. A) Pairwise Pearson correlation coefficients of HPC GSE53987 control samples. Each entry (i, j) represents the Pearson correlation coefficient between the expression of genes i and j along the control samples. The genes are sorted by the SPIN tool (Tsafrir et al., 2005). B) Calculation of a p-value of the observed pairwise correlation coefficient plotted in A). The x-axis represents the P-value thresholds of the pairwise correlation coefficients. The y-axis represents the p-values of the observed pairwise correlation values of the given GWAS-derived gene group, calculated as described in the Supplementary methods section 3. Each “x-number” represents the calculated p-value that corresponds to the threshold represented by the x-axis. C) The same as in A), for PC GSE35978 control samples. D) Calculation of a p-value of the observed pairwise correlation values plotted in C). The plot details are the same as in B).


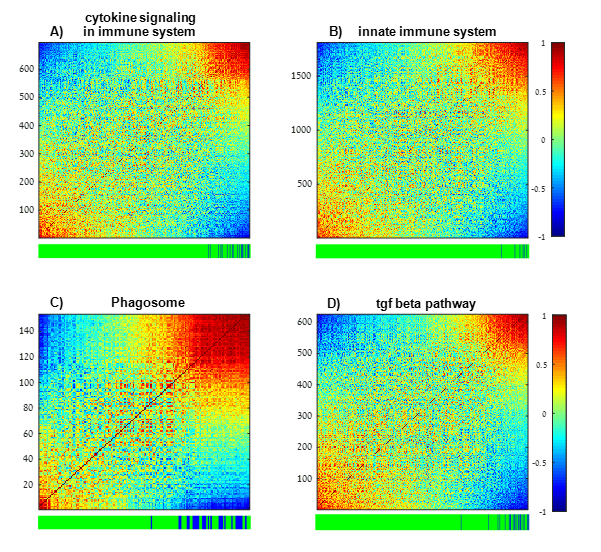


Figure S6: The pairwise Pearson correlation matrix of the proteasome subunits and immune system-related pathways genes**.** Pairwise Pearson correlation matrices were calculated for the GSE53987 dataset for the HPC brain region along MDD samples for the genes composing four immune system-related pathways that were found to be enriched in our analysis. For each pathway, the proteasome subunits genes were added. Each entry (i,j) represents the Pearson correlation coefficient between the expression of genes i and j along the MDD samples. The genes are sorted by the SPIN tool (Tsafrir et al., 2005). A) The pairwise Pearson correlation matrix measured for the genes of the cytokine signaling in immune system pathway and the proteasome subunits. B) The same as in A), for the innate immune system pathway. C) The same as in A), for the phagosome pathway. D) The same as in A), for the tgf-beta pathway.


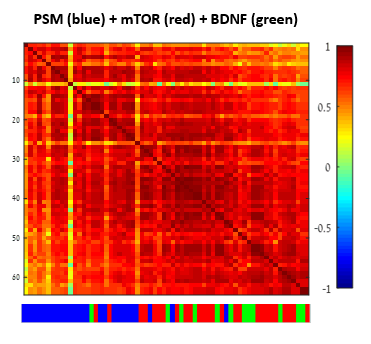


Figure S7: The pairwise Pearson correlation matrix of the proteasome subunits and mTOR+BDNF-related pathway genes. Pairwise Pearson correlation values were calculated for the GSE53987 dataset for the HPC brain region along MDD samples. Each entry (i,j) represents the Pearson correlation coefficient between the expression of genes i and j along the MDD samples. Proteasome (PSM) subunits are marked in blue along the x-axis, mTOR pathway genes in red and BDNF pathway genes in green.


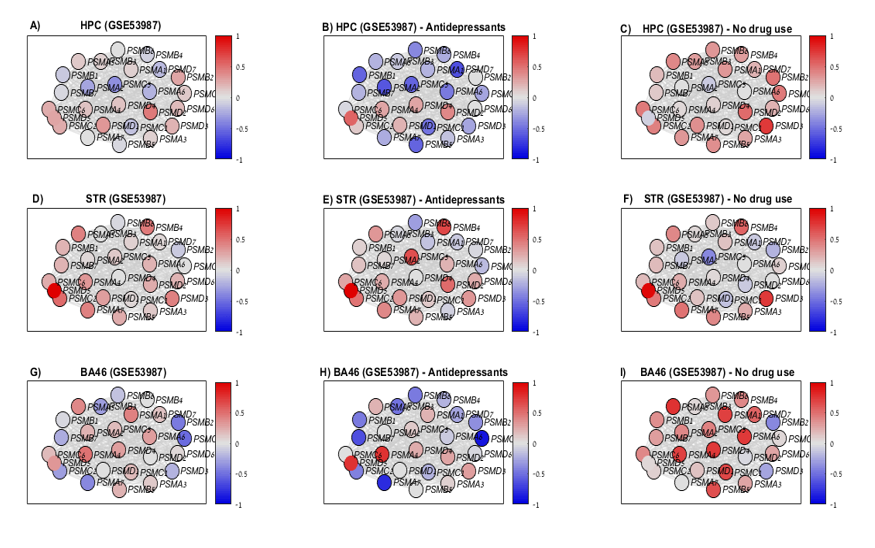
Figure S8: Pathway-based differential expression analysis of the proteasome subunits genes, comparing MDD patients who took antidepressants with those who did not, measured on GSE53987. A) Pathway-based differential expression of the HPC (GSE92538) data. The colors of the nodes represent the deviation in expression of the MDD samples (all of them) from the healthy control group; the edges represent relations of co-expression of the STRING database (See methods section 7). Only genes with a relation to at least one other gene are displayed. B) The same as in A) comparing only MDD patients who took antidepressants to healthy controls. C) The same as in A) comparing only MDD patients who did not take antidepressants to healthy controls. D) The same as in A) for the STR (GSE53987) data. E) The same as in B) for the STR (GSE53987) data. F) The same as in C) for the STR (GSE53987) data. G) The same as in A) for the BA46 (GSE53987) data. H) The same as in B) for the BA46 (GSE53987) data. I) The same as in C) for the BA46 (GSE53987) data.

**Tables**

| **GEO accession** | GSE53987 (Lanz et al., 2015) | | | | | | GSE35978 (Chen et al., 2013) | | | | GSE92538 (Hagenauer et al., 2018) | |
| --- | --- | --- | --- | --- | --- | --- | --- | --- | --- | --- | --- | --- |
| **Brain region** | Hippocampus (HPC) | | Striatum (STR) | | Brodmann area 46 (BA46) | | Parietal Cortex (PC) | | Cerebellum (CRBLM) | | Dorsolateral prefrontal cortex (DLPFC) | |
| **Platform** | Affymetrix Human Genome U133 Plus 2.0 | | | | | | Affymetrix Human Gene 1.0 ST Array | | | | Affymetrix GeneChip Human Genome HG-U133 Plus 2 Array | |
| **Samples** | MDD | CNT | MDD | CNT | MDD | CNT | MDD | CNT | MDD | CNT | MDD | CNT |
| **Samples count** | 16* | 18 | 15** | 18 | 17 | 19 | 14 | 51 | 13 | 50 | 76 | 175 |
| **Gender count** | M=9, F=8 | M=9, F=9 | M=10, F=6 | M=10, F=8 | M=9, F=8 | M=10, F=9 | M=8, F=6 | M=35, F=15 | M=8, F=5 | M=31, F=19 | M=60, F=16 | M=130, F=45 |
| **Mean Age (std)** | 45.17 (10.39) | 48.16 (10.63) | 46.5 (9.64) | 48.44 (10.51) | 45.17 (10.39) | 48.05 (10.36) | 46.07 (9.48) | 45.5 (8.9) | 45.38 (9.49) | 45.8 (9.23) | 48.6 (16.2) | 55.9 (13.6) |
| **Mean PMI (std)** | 20.39 (5.61) | 19.38 (5.05) | 19.41 (6.05) | 19.75 (4.99) | 19.9 (6.03) | 19.52 (4.95) | 27.57 (10.7) | 27.3 (11.7) | 26.38 (10.2) | 27.58 (11.0) | 26 (7.9) | 22.5 (8) |
| **Mean pH (std)** | 6.58 (0.23) | 6.61 (0.2) | 6.57 (0.23) | 6.59 (0.21) | 6.58 (0.23) | 6.59 (0.21) | 6.18 (0.2) | 6.51 (0.3) | 6.25 (0.16) | 6.47 (0.31) | 6.9 (0.3) | 6.8 (0.3) |

Table S1: Expanded description of gene expression data. *One sample was removed from HPC-MDD (GSE53987) as it did not pass quality control (see figure S1A). Of 17 samples, we continued to investigate the remaining 16. **One sample was removed from STR-MDD (GSE53987) as it did not pass quality control (see figure S1B). Of 16 samples, we continued to investigate the remaining 15.

| **QIDs** | **HAM-D** | **RIN** | **Height**  **(m)** | **Weight**  **(Kg)** | **Age (years)** | **F:M** | **N** | **Group** |
| --- | --- | --- | --- | --- | --- | --- | --- | --- |
| Not measured | Not measured | 6.9 | 1.63 ± 0.09 | 60.6 ± 7.9 | 40.6 ± 16.6 | F5:M4 | 9 | Controls |
| 20.6 ± 7.7 | 19.9 ± 6.9 | 6.77 | 1.66 ± 0.14 | 65.4 ± 18.9 | 50.4 ± 8.6 | F7:M2 | 9 | MDD |

Table S2: MDD blood samples cohort. Mean values ± standard deviation values are presented. F, Female; M, Male; RIN, RNA Integrity Number; HAM-D, Hamilton depression rating scale; QIDs, Quick inventory of Depressive symptomatology.

|  | **Score** | **SuperPath Name** | **# SuperPath Total Genes** | **# SuperPath Matched Genes** |
| --- | --- | --- | --- | --- |
| 1 | **11.29** | RAB GEFs Exchange GTP For GDP on RABs | 90 | 3 |
| 2 | **6.45** | Neural Crest Differentiation | 102 | 2 |
| 3 | **5.62** | Initiation of Nuclear Envelope Reformation | 13 | 1 |
| 4 | **5.62** | Development of Pulmonary Dendritic Cells and Macrophage Subsets | 13 | 1 |
| 5 | **5.32** | Deregulation of Rab and Rab Effector Genes in Bladder Cancer | 16 | 1 |
| 6 | **5.15** | Regulation of Wnt/B-catenin Signaling by Small Molecule Compounds | 18 | 1 |
| 7 | **5.15** | Colorectal Cancer Metastasis | 165 | 2 |
| 8 | **4.69** | Glyoxylate Metabolism and Glycine Degradation | 25 | 1 |
| 9 | **4.48** | CDO in Myogenesis | 29 | 1 |
| 10 | **4.34** | Ovarian Infertility Genes | 32 | 1 |
| 11 | **3.96** | Netrin-1 Signaling | 42 | 1 |
| 12 | **3.86** | Citrate Cycle (TCA Cycle) | 45 | 1 |
| 13 | **3.86** | Metal Ion SLC Transporters | 45 | 1 |
| 14 | **3.83** | Histidine, Lysine, Phenylalanine, Tyrosine, Proline and Tryptophan Catabolism | 46 | 1 |
| 15 | **3.77** | Signaling by Slit | 48 | 1 |
| 16 | **3.74** | Netrin Signaling | 49 | 1 |
| 17 | **3.74** | Pyruvate Metabolism and Citric Acid (TCA) Cycle | 49 | 1 |
| 18 | **3.64** | Wnt Signaling Pathway Netpath | 53 | 1 |
| 19 | **3.55** | Vesicle-mediated Transport | 659 | 3 |
| 20 | **3.53** | Coregulation of Androgen Receptor Activity | 57 | 1 |

Table S3: GeneAnalytics pathway enrichment results of the 37 GWAS-based gene group. Superpathway enrichment scores are based on log2-transformation of the binomial p-value, which is equivalent to a p-value corrected for multiple comparisons with significance defined at <0.05. The colors of the score are:
Green (high score): corrected p-value smaller or equal to 0.0001 (Not present in this table).
Yellow (medium score): corrected p-value higher than 0.0001 but smaller or equal to 0.05.
Gray (low score): corrected p-value higher than 0.05.

### **Table S4: GeneAnalytics pathway enrichment results of the 800 extended GWAS-based gene group from the HPC (GSE53987) dataset. - See supplementary tables.**

| **All enriched pathways** | | | | |
| --- | --- | --- | --- | --- |
|  | **800 genes** | **600 genes** | **#intersect** | **Hypergeometric P-value** |
| **PC (GSE35978)** | 134 pathways | 74 pathways | 65 pathways | 1.33×10^-57^ |
| **HPC (GSE53987)** | 120 pathways | 119 pathways | 108 pathways | 1.08×10^-122^ |

### Table S5: Comparison between the resulting GeneAnalytics enriched pathways of the 800 and 600 genes that extended the GWAS-based gene group. The expression levels of these added genes are highly correlated with the average profile of brain regions PC (GSE35978) and HPC (GSE53987) of the GWAS-based gene cluster.

| **Highly scored enriched pathways** | | | | |
| --- | --- | --- | --- | --- |
|  | **800 genes** | **600 genes** | **#intersect** | **Hypergeometric P-value** |
| **PC (GSE35978)** | 76 pathways | 23 pathways | 23 pathways | 0 |
| **HPC (GSE53987)** | 85 pathways | 76 pathways | 71 pathways | 1.13×10^-92^ |

Table S6: Comparison between the highly scored GeneAnalytics enriched pathways that resulted from extending the gene groups by 800 and 600 genes. The expression levels of these added genes are highly correlated with the average profile of brain regions PC (GSE35978) and HPC (GSE53987) of the GWAS-based gene cluster. Highly scored pathways are defined by GeneAnalytics as those with corrected p-values smaller or equal to 0.0001.

### **Table S7: DAVID pathway enrichment results of the 800 extended GWAS-based gene group from the HPC (GSE53987) dataset. - See supplementary tables.**

|  | **Gene symbol** | **Supporting evidence for the involvement of the GWAS-based genes in MDD** |
| --- | --- | --- |
| 1. | NEGR1 | (Mullins and Lewis, 2017)  (Gaspar et al., 2019) (Howard et al., 2019) (Hyde et al., 2016)  (Mullins and Lewis, 2017) |
| 2. | SORCS3 | (Howard et al., 2019)  (Hyde et al., 2016) |
| 3. | SLC30A9 | (Gaspar et al., 2019) |
| 4. | RBFOX1 | (Lin et al., 2018) |
| 5. | RAB27B | (Howard et al., 2019) |
| 6. | RSRC1 | (Howard et al., 2019)  (Hyde et al., 2016) |
| 7. | LRFN5 | (Gaspar et al., 2019) |
| 8. | L3MBTL2 | (Hyde et al., 2016) |
| 9. | TCF4 | (Howard et al., 2019) |
| 10. | DCC | (Mullins and Lewis, 2017)  (Mullins and Lewis, 2017) |
| 11. | OLFM4 | (Hyde et al., 2016) |

Table S8: Supporting evidence for the involvement of the GWAS-based genes in MDD. An in-depth examination demonstrates involvement in MDD of 11 of the 17 genes involved in both clusters of the HPC and PC brain regions (7 genes for HPC, 16 genes for PC; overall 17 genes).

### **Table S9: GeneAnalytics pathway enrichment results of the 800 extended GWAS-based gene group from the PC (GSE35978) dataset. - See supplementary tables.**

### **Table S10: DAVID pathway enrichment results of the 800 extended GWAS-based gene group from the PC (GSE35978) dataset. - See supplementary tables.**

|  | **Genes** | **T-test p-value** | **FDR** | **mean(MDD)/mean(healthy)** |
| --- | --- | --- | --- | --- |
|  |  |  |  |  |
| 1 | 'PMFBP1' | 0.086 | 0.967 | 0.98 |
| 2 | 'SYNE2' | 0.131 | 0.967 | 0.987 |
| 3 | 'RSRC1' | 0.321 | 0.967 | 0.995 |
| 4 | 'TCF4' | 0.327 | 0.967 | 0.997 |
| 5 | 'VRK2' | 0.348 | 0.967 | 0.994 |
| 6 | 'DLST' | 0.452 | 0.967 | 0.999 |
| 7 | 'ASTN2' | 0.537 | 0.967 | 1.001 |
| 8 | 'L3MBTL2' | 0.577 | 0.967 | 1.001 |
| 9 | 'SOX5' | 0.685 | 0.967 | 1.004 |
| 10 | 'NEGR1' | 0.737 | 0.967 | 1.009 |
| 11 | 'DCC' | 0.779 | 0.967 | 1.018 |
| 12 | 'TMEM106B' | 0.78 | 0.967 | 1.004 |
| 13 | 'C6orf168' | 0.791 | 0.967 | 1.012 |
| 14 | 'RAB27B' | 0.806 | 0.967 | 1.01 |
| 15 | 'SLC30A9' | 0.833 | 0.967 | 1.007 |
| 16 | 'DENND1B' | 0.877 | 0.967 | 1.021 |
| 17 | 'CRYBA1' | 0.891 | 0.967 | 1.018 |
| 18 | 'OLFM4' | 0.905 | 0.967 | 1.024 |
| 19 | 'BAG5' | 0.91 | 0.967 | 1.007 |
| 20 | 'SORCS3' | 0.917 | 0.967 | 1.026 |
| 21 | 'RERE' | 0.944 | 0.967 | 1.009 |
| 22 | 'DENND1A' | 0.946 | 0.967 | 1.008 |
| 23 | 'LRFN5' | 0.967 | 0.967 | 1.012 |

### Table S11: Differential expression analysis (two-sided t-test) results for each of the 23 GWAS-based genes of group 1 that are present in GSE53987, comparing HPC MDD samples and controls

|  | **Genes** | **T-test p-value** | **FDR** | **mean(mdd)/mean(healthy)** |
| --- | --- | --- | --- | --- |
|  |  |  |  |  |
| 1 | 'DLST' | 0.013 | 0.357 | 0.989 |
| 2 | 'LINC00461' | 0.032 | 0.447 | 0.956 |
| 3 | 'BAG5' | 0.051 | 0.479 | 0.988 |
| 4 | 'SLC30A9' | 0.121 | 0.528 | 0.988 |
| 5 | 'SYNE2' | 0.124 | 0.528 | 0.979 |
| 6 | 'TMEM106B' | 0.144 | 0.528 | 0.99 |
| 7 | 'TCF4' | 0.158 | 0.528 | 0.994 |
| 8 | 'RSRC1' | 0.175 | 0.528 | 0.992 |
| 9 | 'DENND1B' | 0.183 | 0.528 | 0.991 |
| 10 | 'SORCS3' | 0.198 | 0.528 | 0.988 |
| 11 | 'L3MBTL2' | 0.207 | 0.528 | 0.993 |
| 12 | 'NEGR1' | 0.279 | 0.651 | 0.991 |
| 13 | 'LRFN5' | 0.466 | 0.878 | 0.998 |
| 14 | 'RAB27B' | 0.469 | 0.878 | 0.998 |
| 15 | 'RBFOX1' | 0.5 | 0.878 | 1 |
| 16 | 'SOX5' | 0.502 | 0.878 | 1 |
| 17 | 'TENM2' | 0.6 | 0.899 | 1.006 |
| 18 | 'ENOX1' | 0.62 | 0.899 | 1.003 |
| 19 | 'ASTN2' | 0.639 | 0.899 | 1.003 |
| 20 | 'DCC' | 0.687 | 0.899 | 1.01 |
| 21 | 'SHISA9' | 0.719 | 0.899 | 1.006 |
| 22 | 'DENND1A' | 0.726 | 0.899 | 1.004 |
| 23 | 'OLFM4' | 0.739 | 0.899 | 1.007 |
| 24 | 'CRYBA1' | 0.8 | 0.911 | 1.007 |
| 25 | 'TOPAZ1' | 0.842 | 0.911 | 1.008 |
| 26 | 'PMFBP1' | 0.865 | 0.911 | 1.013 |
| 27 | 'VRK2' | 0.879 | 0.911 | 1.034 |
| 28 | 'RERE' | 0.971 | 0.971 | 1.008 |

### Table S12: Differential expression analysis (two-sided t-test) results for each of the 28 GWAS-based genes of group 1 that are present in GSE35978, comparing PC MDD samples and controls

|  | **STR (GSE53987)** | **HPC (GSE53987)** | **B46 (GSE53987)** | **PC (GSE35978)** | **CRBLM (GSE35978)** | **DLPFC (GSE92538)** | **CRBLM (Stanely #6)** | **Thalamus (Stanely #16)** |
| --- | --- | --- | --- | --- | --- | --- | --- | --- |
| **STR** | 1 (0) | 0.140 (2.4×10^-91^) | 0.120 (1.4×10^-67^) | -0.112 (4.5×10^-41^) | 0.058 (6.3×10^-12^) | 0.140 (3.4×10^-45^) | 0.121 (1.1×10^-25^) | -0.310 (6.4×10^-318^) |
| **HPC** | 0.140 (2.4×10^-91^) | 1 (0) | 0.031 (6.3×10^-06^) | -0.072 (1.2×10^-17^) | 0.041 (1×10^-6^) | 0.247 (3.6×10^-139^) | 0.001 (0.936) | -0.106 (2.4×10^-37^) |
| **B46** | 0.120 (1.4×10^-67^) | 0.031 (6.3×10^-06^) | 1 (0) | -0.017 (0.049) | 0.085 (2.7×10^-24^) | 0.145 (5.2×10^-48^) | -0.006 (0.589) | -0.102 (2.1×10^-34^) |
| **PC** | -0.112 (4.5×10^-41^) | -0.072 (1.2×10^-17^) | -0.017 (0.049) | 1 (0) | -0.001 (0.841) | -0.393 (0) | 0.051 (3×10^-6^) | 0.418 (0) |
| **CRBLM** | 0.058 (6.3×10^-12^) | 0.041 (1×10^-6^) | 0.085 (2.7×10^-24^) | -0.001 (0.841) | 1 (0) | 0.071 (2.5×10^-14^) | 0.014 (0.186) | -0.100 (1.2×10^-40^) |
| **DLPFC** | 0.140 (3.4×10^-45^) | 0.247 (3.6×10^-139^) | 0.145 (5.2×10^-48^) | -0.393 (0) | 0.071 (2.5×10^-14^) | 1 (0) | -0.003 (0.792) | -0.540 (0) |
| **CRBLM**  **( Stanely #6)** | 0.121 (1.1×10^-25^) | 0.001 (0.936) | -0.006 (0.589) | 0.051 (3×10^-6^) | 0.014 (0.186) | -0.003 (0.792) | 1 (0) | -0.047 (1.5×10^-5^) |
| **Thalamus**  **(Stanely #16)** | -0.310 (6.4×10^-318^) | -0.106 (2.4×10^-37^) | -0.102 (2.1×10^-34^) | 0.418 (0) | -0.100 (1.2×10^-40^) | -0.540 (0) | -0.047 (1.5×10^-5^) | 1 (0) |

Table S13: Pairwise dataset comparability measures for three MDD gene expression datasets, applied to each region separately. Each entry (i.j) represents the t-statistic (MDD vs. controls) Pearson correlation value (rho P-value) along all common genes between the dataset in row i and the dataset in column j. The correlation values marked in blue are of the largest group, with a mutual large positive correlation (defined as rho >1 and p-value < 0.001).

| **#** | **Gene symbol** | **Description** | **Fold Change** | **p-value** | **Corrected p-value** |
| --- | --- | --- | --- | --- | --- |
| 1 | PSMB5 | proteasome 20S subunit beta 5 | 1.359302 | 0.002433 | 0.128946 |
| 2 | PSMA3-AS1 | PSMA3 antisense RNA 1 | 1.166946 | 0.021638 | 0.573394 |
| 3 | PSMD5 | proteasome 26S subunit, non-ATPase 5 | 1.20216 | 0.049936 | 0.630137 |
| 4 | PSMC2 | proteasome 26S subunit, ATPase 2 | 1.153683 | 0.050519 | 0.630137 |
| 5 | PSMD13 | proteasome 26S subunit, non-ATPase 13 | 1.100735 | 0.083681 | 0.630137 |
| 6 | PSMC3IP | PSMC3 interacting protein | 1.158512 | 0.103055 | 0.630137 |
| 7 | PSME3 | proteasome activator subunit 3 | 1.100645 | 0.103507 | 0.630137 |
| 8 | PSMB10 | proteasome 20S subunit beta 10 | 0.854651 | 0.111539 | 0.630137 |
| 9 | PSME3IP1 | proteasome activator subunit 3 interacting protein 1 | 1.140713 | 0.115576 | 0.630137 |
| 10 | PSMC4 | proteasome 26S subunit, ATPase 4 | 0.922839 | 0.118894 | 0.630137 |

Table S14: Top 10 differentially expressed proteasome subunits genes between PBMCs of 9 MDD patients and 9 healthy controls. Correction for multiple comparisons was performed using the Benjamini and Hochberg false discovery rate (FDR) method (Benjamini and Hochberg, 1995).

### References

Benjamini, Y., Hochberg, Y., 1995. Controlling the False Discovery Rate: A Practical and Powerful Approach to Multiple Testing. Journal of the Royal Statistical Society 57, 289–300.

Chen, C., Cheng, L., Grennan, K., Pibiri, F., Zhang, C., Badner, J.A., Members of the Bipolar Disorder Genome Study, C., Gershon, E.S., Liu, C., 2013. Two gene co-expression modules differentiate psychotics and controls. Mol Psychiatry 18, 1308-1314.

Gaspar, H.A., Gerring, Z., Hubel, C., Major Depressive Disorder Working Group of the Psychiatric Genomics, C., Middeldorp, C.M., Derks, E.M., Breen, G., 2019. Using genetic drug-target networks to develop new drug hypotheses for major depressive disorder. Transl Psychiatry 9, 117.

Hagenauer, M.H., Schulmann, A., Li, J.Z., Vawter, M.P., Walsh, D.M., Thompson, R.C., Turner, C.A., Bunney, W.E., Myers, R.M., Barchas, J.D., Schatzberg, A.F., Watson, S.J., Akil, H., 2018. Inference of cell type content from human brain transcriptomic datasets illuminates the effects of age, manner of death, dissection, and psychiatric diagnosis. PLoS One 13, e0200003.

Howard, D.M., Adams, M.J., Clarke, T.K., Hafferty, J.D., Gibson, J., Shirali, M., Coleman, J.R.I., Hagenaars, S.P., Ward, J., Wigmore, E.M., Alloza, C., Shen, X., Barbu, M.C., Xu, E.Y., Whalley, H.C., Marioni, R.E., Porteous, D.J., Davies, G., Deary, I.J., Hemani, G., Berger, K., Teismann, H., Rawal, R., Arolt, V., Baune, B.T., Dannlowski, U., Domschke, K., Tian, C., Hinds, D.A., andMe Research, T., Major Depressive Disorder Working Group of the Psychiatric Genomics, C., Trzaskowski, M., Byrne, E.M., Ripke, S., Smith, D.J., Sullivan, P.F., Wray, N.R., Breen, G., Lewis, C.M., McIntosh, A.M., 2019. Genome-wide meta-analysis of depression identifies 102 independent variants and highlights the importance of the prefrontal brain regions. Nat Neurosci 22, 343-352.

Hyde, C.L., Nagle, M.W., Tian, C., Chen, X., Paciga, S.A., Wendland, J.R., Tung, J.Y., Hinds, D.A., Perlis, R.H., Winslow, A.R., 2016. Identification of 15 genetic loci associated with risk of major depression in individuals of European descent. Nat Genet 48, 1031-1036.

Irizarry, R.A., Hobbs, B., Collin, F., Beazer-Barclay, Y.D., Antonellis, K.J., Scherf, U., Speed, T.P., 2003. Exploration, normalization, and summaries of high density oligonucleotide array probe level data. Biostatistics 4, 249-264.

Kampe, A., Enlund-Cerullo, M., Valkama, S., Holmlund-Suila, E., Rosendahl, J., Hauta-Alus, H., Pekkinen, M., Andersson, S., Makitie, O., 2019. Genetic variation in GC and CYP2R1 affects 25-hydroxyvitamin D concentration and skeletal parameters: A genome-wide association study in 24-month-old Finnish children. PLoS Genet 15, e1008530.

Lanz, T.A., Joshi, J.J., Reinhart, V., Johnson, K., Grantham, L.E., 2nd, Volfson, D., 2015. STEP levels are unchanged in pre-frontal cortex and associative striatum in post-mortem human brain samples from subjects with schizophrenia, bipolar disorder and major depressive disorder. PLoS One 10, e0121744.

Le, K.T.T., Matzaraki, V., Netea, M.G., Wijmenga, C., Moser, J., Kumar, V., 2019. Functional Annotation of Genetic Loci Associated With Sepsis Prioritizes Immune and Endothelial Cell Pathways. Front Immunol 10, 1949.

Lin, E., Kuo, P.H., Liu, Y.L., Yu, Y.W., Yang, A.C., Tsai, S.J., 2018. A Deep Learning Approach for Predicting Antidepressant Response in Major Depression Using Clinical and Genetic Biomarkers. Front Psychiatry 9, 290.

Mullins, N., Lewis, C.M., 2017. Genetics of Depression: Progress at Last. Curr Psychiatry Rep 19, 43.

Petersen, A., Alvarez, C., DeClaire, S., Tintle, N.L., 2013. Assessing methods for assigning SNPs to genes in gene-based tests of association using common variants. PLoS One 8, e62161.

Tsafrir, D., Tsafrir, I., Ein-Dor, L., Zuk, O., Notterman, D.A., Domany, E., 2005. Sorting points into neighborhoods (SPIN): data analysis and visualization by ordering distance matrices. Bioinformatics 21, 2301-2308.

Wray, N.R., Ripke, S., Mattheisen, M., Trzaskowski, M., Byrne, E.M., Abdellaoui, A., Adams, M.J., Agerbo, E., Air, T.M., Andlauer, T.M.F., Bacanu, S.A., Baekvad-Hansen, M., Beekman, A.F.T., Bigdeli, T.B., Binder, E.B., Blackwood, D.R.H., Bryois, J., Buttenschon, H.N., Bybjerg-Grauholm, J., Cai, N., Castelao, E., Christensen, J.H., Clarke, T.K., Coleman, J.I.R., Colodro-Conde, L., Couvy-Duchesne, B., Craddock, N., Crawford, G.E., Crowley, C.A., Dashti, H.S., Davies, G., Deary, I.J., Degenhardt, F., Derks, E.M., Direk, N., Dolan, C.V., Dunn, E.C., Eley, T.C., Eriksson, N., Escott-Price, V., Kiadeh, F.H.F., Finucane, H.K., Forstner, A.J., Frank, J., Gaspar, H.A., Gill, M., Giusti-Rodriguez, P., Goes, F.S., Gordon, S.D., Grove, J., Hall, L.S., Hannon, E., Hansen, C.S., Hansen, T.F., Herms, S., Hickie, I.B., Hoffmann, P., Homuth, G., Horn, C., Hottenga, J.J., Hougaard, D.M., Hu, M., Hyde, C.L., Ising, M., Jansen, R., Jin, F., Jorgenson, E., Knowles, J.A., Kohane, I.S., Kraft, J., Kretzschmar, W.W., Krogh, J., Kutalik, Z., Lane, J.M., Li, Y., Li, Y., Lind, P.A., Liu, X., Lu, L., MacIntyre, D.J., MacKinnon, D.F., Maier, R.M., Maier, W., Marchini, J., Mbarek, H., McGrath, P., McGuffin, P., Medland, S.E., Mehta, D., Middeldorp, C.M., Mihailov, E., Milaneschi, Y., Milani, L., Mill, J., Mondimore, F.M., Montgomery, G.W., Mostafavi, S., Mullins, N., Nauck, M., Ng, B., Nivard, M.G., Nyholt, D.R., O'Reilly, P.F., Oskarsson, H., Owen, M.J., Painter, J.N., Pedersen, C.B., Pedersen, M.G., Peterson, R.E., Pettersson, E., Peyrot, W.J., Pistis, G., Posthuma, D., Purcell, S.M., Quiroz, J.A., Qvist, P., Rice, J.P., Riley, B.P., Rivera, M., Saeed Mirza, S., Saxena, R., Schoevers, R., Schulte, E.C., Shen, L., Shi, J., Shyn, S.I., Sigurdsson, E., Sinnamon, G.B.C., Smit, J.H., Smith, D.J., Stefansson, H., Steinberg, S., Stockmeier, C.A., Streit, F., Strohmaier, J., Tansey, K.E., Teismann, H., Teumer, A., Thompson, W., Thomson, P.A., Thorgeirsson, T.E., Tian, C., Traylor, M., Treutlein, J., Trubetskoy, V., Uitterlinden, A.G., Umbricht, D., Van der Auwera, S., van Hemert, A.M., Viktorin, A., Visscher, P.M., Wang, Y., Webb, B.T., Weinsheimer, S.M., Wellmann, J., Willemsen, G., Witt, S.H., Wu, Y., Xi, H.S., Yang, J., Zhang, F., eQtlgen, andMe, Arolt, V., Baune, B.T., Berger, K., Boomsma, D.I., Cichon, S., Dannlowski, U., de Geus, E.C.J., DePaulo, J.R., Domenici, E., Domschke, K., Esko, T., Grabe, H.J., Hamilton, S.P., Hayward, C., Heath, A.C., Hinds, D.A., Kendler, K.S., Kloiber, S., Lewis, G., Li, Q.S., Lucae, S., Madden, P.F.A., Magnusson, P.K., Martin, N.G., McIntosh, A.M., Metspalu, A., Mors, O., Mortensen, P.B., Muller-Myhsok, B., Nordentoft, M., Nothen, M.M., O'Donovan, M.C., Paciga, S.A., Pedersen, N.L., Penninx, B., Perlis, R.H., Porteous, D.J., Potash, J.B., Preisig, M., Rietschel, M., Schaefer, C., Schulze, T.G., Smoller, J.W., Stefansson, K., Tiemeier, H., Uher, R., Volzke, H., Weissman, M.M., Werge, T., Winslow, A.R., Lewis, C.M., Levinson, D.F., Breen, G., Borglum, A.D., Sullivan, P.F., Major Depressive Disorder Working Group of the Psychiatric Genomics, C., 2018. Genome-wide association analyses identify 44 risk variants and refine the genetic architecture of major depression. Nat Genet 50, 668-681.

Zhang, Y.B., Hu, J., Zhang, J., Zhou, X., Li, X., Gu, C., Liu, T., Xie, Y., Liu, J., Gu, M., Wang, P., Wu, T., Qian, J., Wang, Y., Dong, X., Yu, J., Zhang, Q., 2016. Genome-wide association study identifies multiple susceptibility loci for craniofacial microsomia. Nat Commun 7, 10605.
